# Impact of Sex Differences on Mortality in Patients With Sepsis After Trauma: A Nationwide Cohort Study

**DOI:** 10.3389/fimmu.2021.678156

**Published:** 2021-06-29

**Authors:** Yutaka Kondo, Atsushi Miyazato, Ken Okamoto, Hiroshi Tanaka

**Affiliations:** Department of Emergency and Critical Care Medicine, Juntendo University Urayasu Hospital, Chiba, Japan

**Keywords:** sex, female, trauma, sepsis, mortality

## Abstract

**Objective:**

Sepsis is the leading cause of death in intensive care units, and sepsis after trauma is associated with increased mortality rates. However, the characteristics of sepsis after trauma remain unknown, and the influence of sex on mortality remains controversial. This study aimed to assess the role of sex in in-hospital mortality in patients with sepsis after trauma.

**Methods:**

We performed a retrospective cohort study involving several emergency hospitals (n=288) in Japan. The data of patients with trauma who developed sepsis after admission from 2004 to 2019 were obtained from the Japan Trauma Data Bank. We divided the patients into two groups according to sex and compared their in-hospital mortality. We also performed subgroup analysis limited to the elderly population (age ≥ 65 years) and evaluated in-hospital mortality between men and women.

**Results:**

A total of 1935 patients met the inclusion criteria during the study period. Of these, 1204 (62.2%) were allocated to the male group and 731 (37.8%) to the female group. Multivariable Cox proportional-hazards analysis showed a significantly lower risk of in-hospital mortality in the female group than in the male group (hazard ratio (HR): 0.74, 95% confidence interval (CI): 0.62–0.89; p=0.001). In the subgroup analysis, multivariable Cox proportional hazards still showed significantly lower risks of in-hospital mortality in the female group than in the male group (HR: 0.72, 95% CI: 0.58–0.88; p=0.002).

**Conclusion:**

The present study shows a significantly increased survival in the female group when compared to that in the male group of patients with sepsis after trauma. The underlying mechanism remains unclear, and further investigations are required.

## Introduction

Sepsis is the leading cause of death in intensive care units (ICUs) and accounts for more than five million deaths worldwide each year (1, 2). Moreover, sepsis-associated mortality increases after trauma because of injuries, surgeries, adverse events, and complications, which weaken the immune system. Therefore, a fundamental understanding of sepsis following injury is important to improve clinical outcomes.

Biological sex is known to modulate inflammatory responses during immune processes (3). Several studies using animal models have shown that the sex hormones testosterone and estrogen play a central role in the regulation of posttraumatic immunosuppression (4–6). Testosterone depletion and estrogen supplemental therapy can improve mortality (6). Furthermore, non-hormonal, genetic factor related sexual dimorphism also account for differences in incidence or clinical manifestations of autoimmune diseases, malignancies, and infection (7).

In contrast, the influence of sex on mortality in patients with sepsis remains controversial in clinical settings. Several studies have reported that mortality is lower in women with sepsis than in men with sepsis (8, 9) whereas others have reported increased mortality among women (10, 11). Thus, the influence of sex on mortality in patients with sepsis after trauma remains unknown. We hypothesized that being female sex is associated with lower mortality in cases of sepsis after trauma. This study aimed to elucidate how sex affects in-hospital mortality in patients with sepsis after trauma using a nationwide Japanese cohort dataset.

## Materials and Methods

The present study was approved by the Ethics Committee of the Juntendo University Urayasu Hospital. The requirement for informed consent was waived because of the anonymous nature of the data set. This study followed the STROBE statement (12).

### Study Design and Data Collection

This was a multicenter retrospective cohort study using data from patients treated for sepsis after trauma between 2004 and 2019. The data were available at the Japan Trauma Data Bank (JTDB), which was established in 2003 by the Japanese Association for the Surgery of Trauma (Trauma Registry Committee) and the Japanese Association for Acute Medicine (Committee for Clinical Care Evaluation) as the main parties (13). The database contains nationwide data on patients with trauma and includes patient characteristics, vital signs during the prehospital phase and upon hospital arrival, examinations and treatment records, types and causes of trauma, diagnostic codes using the abbreviated injury scale score (14), several trauma severity scores, hospitalization length, and discharge status (15).

The data derived from 288 emergency hospitals in Japan, which were voluntarily enrolled in the study. The database contains data from approximately 71% of tertiary-level emergency hospitals in Japan (16). Approximately 40% of the participating hospitals are comparable to level I trauma centers in the United States (17, 18).

### Study Participants

We included trauma patients who developed sepsis after admission. The definition of sepsis is “sepsis with multiple organ failure”, which is equivalent to “severe sepsis” in the conventional systemic inflammatory response syndrome criteria (Sepsis-1 or -2) (19), or current “sepsis” criteria (Sepsis-3) proposed at 2016 (20). Patients lacking sex and systolic blood pressure data or whose systolic blood pressure was zero upon arrival were excluded.

Eligible patients were divided into two groups: male and female.

### Variables and Outcomes

In this study, we examined the following patient characteristics: age, sex, vital signs upon arrival, cause of injury, injury severity score (ISS), type of trauma (blunt, penetrating, burn, or others), cause of trauma (accident, suicide attempt, or others), admission year, treatment (resuscitative endovascular balloon occlusion of the aorta, transcatheter arterial embolization, or surgical operation), hospitalization length, and in-hospital mortality.

The primary outcome in this study was in-hospital mortality among men and women with sepsis after trauma.

### Statistical Analysis

Continuous variables are presented as median and interquartile ranges. Categorical variables appear as numbers and percentages. Baseline characteristics and crude outcomes were compared using the Mann-Whitney U test for the skewed distribution of continuous variables and the chi-square test or Fisher’s exact test for categorical variables.

We performed multivariable Cox proportional hazards regression analysis for the time until death during hospitalization and adjusted for all the variables mentioned above (21). Hazard ratios (HRs) and the associated 95% confidence intervals (CIs) were calculated for in-hospital mortality. For sensitivity analysis, we have added a traumatic brain injury variable which showed head AIS≥1 for adjustment.

We also performed subgroup analysis using the multivariable Cox proportional hazards regression method. The variables used in the main analysis were also used in the subgroup analysis, which involved patients aged ≥65 years only because the effects of sex hormones are attenuated in the elderly.

The two-sided significance level for all tests was set at p <.05. All analyses were performed using IBM SPSS, version 26 (IBM Corp., Armonk, NY, USA).

## Results

**Figure 1:**
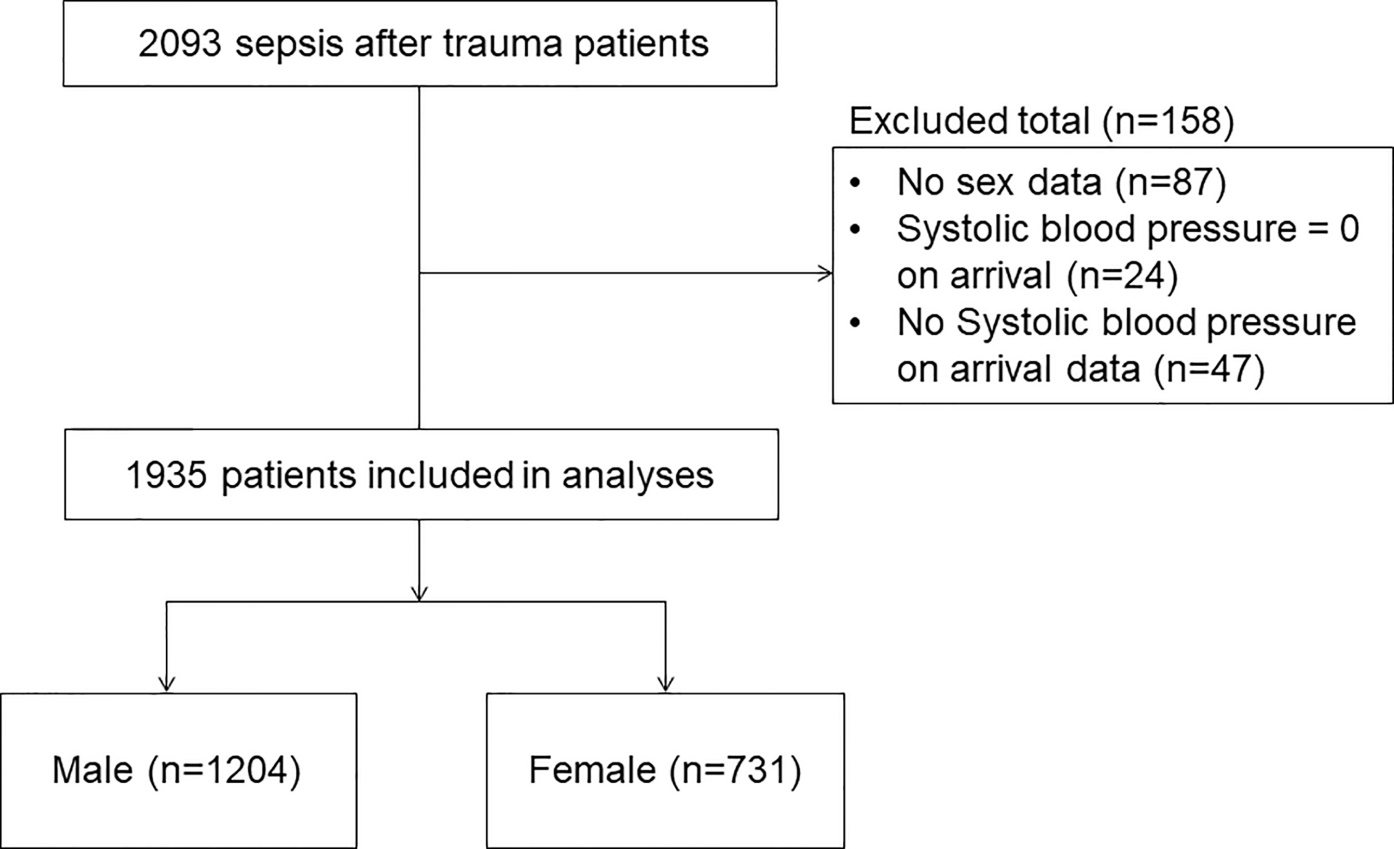
Flow diagram showing the process for selecting patients.

A total of 1935 patients met the inclusion criteria during the study period. Of these, 1204 (62.2%) were allocated to the male group and 731 (37.8%) to the female group (**Figure 1**).

The baseline characteristics and outcomes are shown in **Table 1**. The median age and temperature were higher in women than in men. The median heart rate, respiratory rate, and ISS were lower in women than in men. Glasgow Coma Scale scores and causes of trauma were significantly different between the groups. There were no significant differences in systolic blood pressure, type of trauma, year, or treatment between men and women. Regarding outcomes, the median length of hospitalization was 36.5 days in women and 44 days in men. Crude in-hospital mortality was 36.5% among women and 47.4% among men.

**Table 1:** Baseline characteristics of eligible patients.

	Male	Female	*p*-value	Number of patients with missing data, n (%)
	(n = 1204)	(n = 731)		
Age, years (range)	68 (53–80)	79 (65–85)	<0.001	2 (0.10)
Systolic blood pressure, mmHg	129 (97-156)	132 (105-157)	0.056	0 (0)
Heart rate, beats/min	93 (76-112)	89 (75-104)	0.004	37 (1.9)
Respiratory rate, breaths/min	22 (18-28)	21 (18-26)	0.009	164 (8.5)
Temperature, °C	36.2 (35.6)	36.4 (36.8)	0.001	204 (10.5)
GCS			0.002	111 (5.7)
3-5	119 (10.4)	60 (3.8)		
6-8	129 (11.3)	56 (8.2)		
9-11	116 (10.2)	44 (6.4)		
12-14	325 (28.5)	215 (31.4)		
15	451 (39.6)	309 (45.2)		
ISS	25 (16–29)	16 (9–25)	<0.001	10 (0.52)
Type of trauma			0.78	11 (0.57)
Blunt	906 (75.8)	555 (76.2)		
Penetrating	22 (1.8)	16 (2.2)		
Burn	253 (21.2)	151 (20.7)		
Others	15 (1.3)	6 (0.8)		
Cause of trauma			<0.001	13 (0.67)
Accident	915 (76.6)	616 (84.7)		
Suicide attempt	103 (8.6)	79 (10.9)		
Others	177 (14.8)	32 (4.4)		
Year			0.41	8 (0.41)
2004–2007	91 (7.6)	55 (7.6)		
2008–2011	342 (28.5)	192 (26.4)		
2012–2015	507 (42.3)	335 (46.1)		
2016–2019	260 (21.7)	145 (19.9)		
REBOA	35 (2.9)	17 (2.3)	0.44	0 (0)
TAE	142 (11.8)	73 (10.0)	0.22	0 (0)
Surgical operation	858 (71.3)	546 (74.7)	0.10	0 (0)
Hospitalization length, days	44 (19–82)	36.5 (19–68)	0.012	32 (1.7)
In-hospital mortality	556 (47.4)	260 (36.5)	<0.001	50 (2.6)

Data are presented as number (%) or median (interquartile range).

GCS, Glasgow coma scale; ISS, injury severity score; REBOA, resuscitative endovascular balloon occlusion of the aorta; TAE, transcatheter arterial embolization.

The number of patients with missing data is shown in **Table 1**.

**Figure 2:**
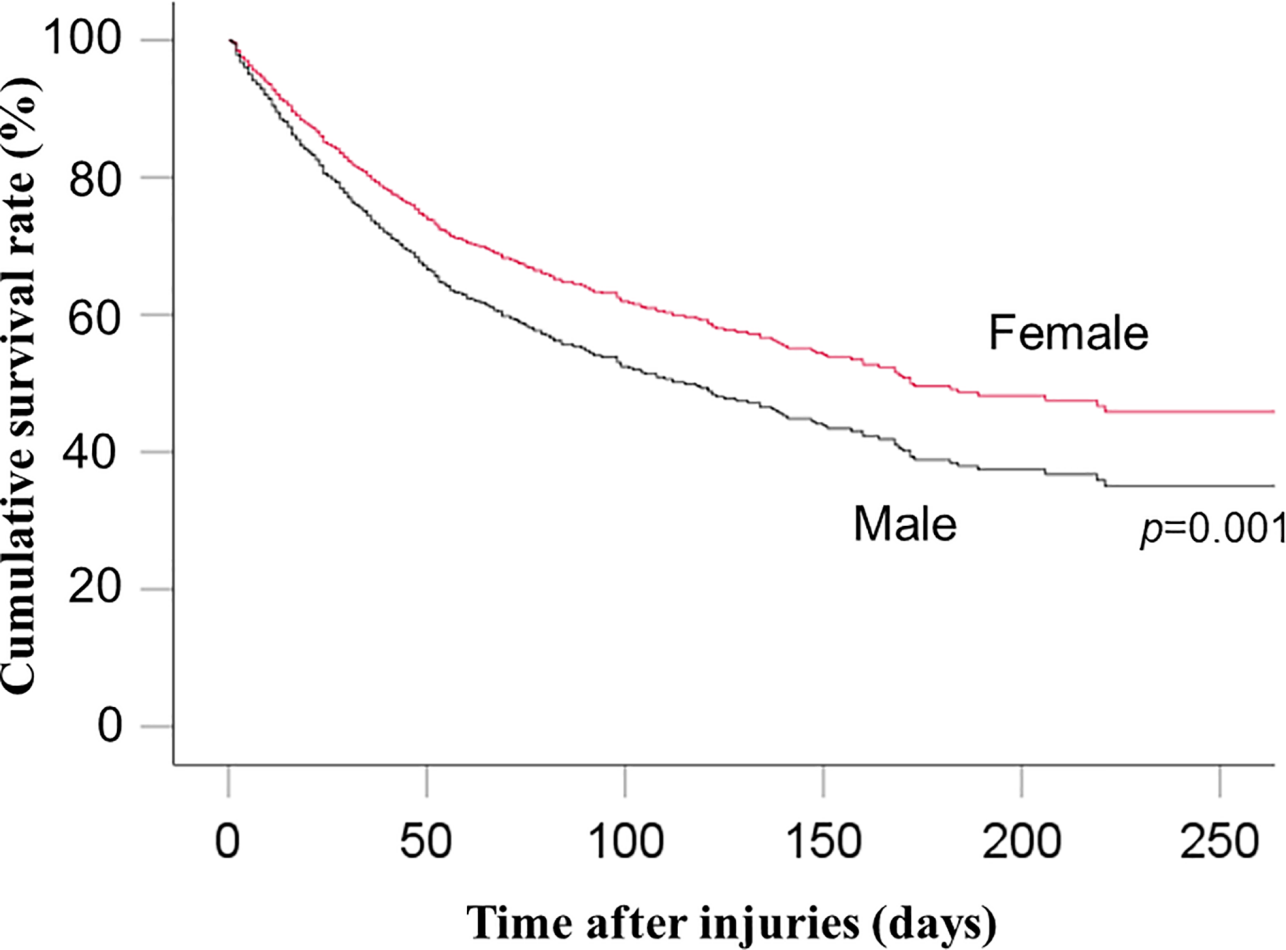
Cumulative survival rate between men and women.

Multivariable Cox proportional hazards analysis showed significantly lower risk of in-hospital mortality among women than among men (HR: 0.74, 95% CI: 0.62–0.89; p=0.001) (**Table 2** and **Figure 2**). Sensitivity analysis also showed significantly lower risk of in-hospital mortality among women than among men (HR: 0.76, 95% CI: 0.63–0.91; p=0.003) (**Supplemental File 1**).

**Table 2:** Cox proportional hazards regression for in-hospital mortality.

	Hazard ratio	95% confidence interval	*p*-value
Sex			
Male	Reference		
Female	0.74	0.62-0.89	0.001
Age, by 1-year increase	1.02	1.02-1.03	<0.001
Systemic blood pressure, by 1-mmHg increase	0.99	0.99-1.00	0.024
Heart rate, by 1-beat/min increase	0.99	0.99-1.00	0.37
Respiratory rate, by 1-time/min increase	1.02	1.01-1.03	0.002
Temperature, by 1-centigrade increase	0.91	0.86-0.96	0.001
GCS			
3-5	Reference		
6-8	0.68	0.49-0.95	0.025
9-11	0.54	0.37-0.77	0.001
12-14	0.55	0.42-0.73	<0.001
15	0.48	0.36-0.64	<0.001
ISS, by 1-point increase	1.01	1.01-1.02	<0.001
Type of trauma			
Blunt	Reference		
Penetrating	1.10	0.55-2.19	0.78
Burn	1.96	1.61-2.40	<0.001
Others	0.91	0.36-2.31	0.84
Cause of trauma			
Accident	Reference		
Suicide attempt	1.25	0.93-1.70	0.14
Others	1.07	0.80-1.42	0.65
Year			
2004–2007	Reference		
2008–2011	0.56	0.41-0.75	<0.001
2012–2015	0.54	0.41-0.73	<0.001
2016–2019	0.50	0.37-0.69	<0.001
REBOA	0.79	0.45-1.38	0.40
TAE	1.13	0.85-1.50	0.40
Surgical operation	0.43	0.36-0.52	<0.001

GCS, Glasgow coma scale; ISS, injury severity score; REBOA, resuscitative endovascular balloon occlusion of the aorta; TAE, transcatheter arterial embolization.

Subgroup analysis included 1237 elderly patients (≥65 years of age). Multivariable Cox proportional-hazards analysis showed a significantly lower risk of in-hospital mortality in women than in men (HR: 0.72, 95% CI: 0.58–0.88; p=0.002) (**Supplemental File 2**).

## Discussion

The present nationwide retrospective cohort study showed that the in-hospital mortality in men with sepsis after trauma is significantly higher than in women. In our subgroup analysis on elderly patients, in-hospital mortality among women was also lower than that among men. Therefore, the main results are in line with our hypothesis. Similar results were shown in sensitivity analysis.

There is limited evidence on the role of sex in sepsis after trauma. In 2000, an observational study found no difference in the mortality rates among men and women (men 64.9%, women 65.5%) with severe sepsis or septic shock in surgical patients (22). In 2014, another observational study reported that female sex was associated with improved clinical outcomes in cases of sepsis and organ failure after traumatic injury and hemorrhagic shock (23). Our findings are in line with the results of these studies. However, studies assessing several clinical outcomes are required because our study evaluated only in-hospital mortality in men and women.

Several studies have reported that mortality among women with trauma and hemorrhagic shock was significantly lower than in men (24–26) although the underlying reasons have not been fully elucidated. In 2019, an observational study showed that women were more likely to have a hypercoagulable profile when compared to men, and consequently were less likely to develop traumatic coagulopathy after severe trauma (24). Furthermore, the association between hypercoagulability and female hormones has been previously reported (27, 28) and the association between estrogen and the clotting cascade in trauma patients has been confirmed in a previous study (29). Altogether, these data suggest that hypercoagulability in women contributes to reducing mortality due to trauma. Similarly, in our study, hypercoagulability in women might have also contributed to decreasing in-hospital mortality in patients with sepsis after trauma.

Some studies consider that hormonal differences are the primary underlying cause of the mortality differences between men and women (30, 31). Moreover, a study suggested that younger women had lower mortality, unlike elderly women due to the age-dependent hormonal status (30). To evaluate the effects of sex hormones on in-hospital mortality, we performed a subgroup analysis including exclusively elderly patients. If the difference in in-hospital mortality between men and women were strongly dependent on female hormones, the mortality among postmenopausal women might be higher than that in young women, and might not significantly differ from that of men. However, subgroup analysis shows that the in-hospital mortality in elderly women was lower than that of elderly men. This result implies that multiple factors are involved in in-hospital mortality in addition to hormonal differences. In line with this observation, several studies have reported that the treatment and care provided at hospitals differ between male and female patients (32, 33). Alternatively, high estrogen levels throughout a woman’s life may be associated with health benefits even after menopause. Another possible explanation is non-hormonal, genetic factor related sexual dimorphism; several genes encoding for innate immune molecules are located on the X chromosome (7). In fact, an animal study reported that X-linked genetic variations in genes involved in the immune response were associated with differences in defense against infections between males and females (34).

Pathogen-associated molecular patterns (PAMPs) and damage-associated molecular patterns (DAMPs) play an important role in sepsis as potent activators of initiating innate immune system (35, 36). Inflammation, organ injury, and death that follows infection were due to the body’s response to PAMPs and DAMPs. This mechanism of immune system may differ in sex and may lead to different mortality rate.

Our study showed that the female group was older than the male group. The male group had higher ISS scores than the female group. These findings might come from different patterns of trauma between the groups (37). We therefore used the variables, age, ISS scores, and cause of trauma, for main analysis and still got lower mortality rate in the female group even after adjustment.

We excluded following patients with no sex data because sex data is essential for the design of present study. We also excluded systolic blood pressure was zero on arrival and no systolic blood pressure data because the number of was small, and clinical characteristics differ between cardiac arrest before arrival and the others.

There are several limitations in this study. First, the definition of sepsis changes over time and may affect sepsis-associated mortality data assessments. Second, some data on the evaluated variables were missing and excluded from our analysis, which may have affected the main results. Third, we could not perform sample size calculation because differences of anticipated mortality rate between the groups are unknown. However, we have used a large database and therefore statistically underpower is unlikely. Furthermore, sample size calculation is mostly required prospective design because it needs to determine study period and number of included patients. Our study is retrospective design and includes long study period and many patients. Finally, we have selected independent variables by clinical importance. We retrospectively analyzed the data, and some unadjusted confounders might have been overlooked. Selection of variables could affect results although our sensitivity analysis supported main results.

## Conclusion

The present retrospective cohort study contained data from hospitals throughout Japan and showed that mortality due to sepsis after trauma was significantly lower among females than among males. However, further studies are required to unveil the underlying causes of the sex-associated mortality differences observed in this study.

## Data Availability Statement

The data analyzed in this study is subject to the following licenses/restrictions: Data are available upon reasonable request although data are not in public. Requests to access these datasets should be directed to YK, kondokondou2000@yahoo.co.jp.

## Ethics Statement

The studies involving human participants were reviewed and approved by the Ethics Committee of the Juntendo University Urayasu Hospital. Written informed consent from the participants’ legal guardian/next of kin was not required to participate in this study in accordance with the national legislation and the institutional requirements.

## Author Contributions

YK conceived this study, analyzed the data, and drafted the manuscript. AM partially revised the manuscript. KO and HT advised on the design and revised the manuscript. All authors contributed to the article and approved the submitted version.

## Conflict of Interest

The authors declare that the research was conducted in the absence of any commercial or financial relationships that could be construed as a potential conflict of interest.
