# Supplementary material for: Impact of Sex Differences on Mortality in Patients With Sepsis After Trauma: A Nationwide Cohort Study
Source: Front Immunol. 2021 Jun 29;12:678156. doi: 10.3389/fimmu.2021.678156 (PMC8276106; doi:10.3389/fimmu.2021.678156)
Supplement: Supplementary file 1 [file DataSheet_1.pdf]

Supplemental file 1. Sensitivity analysis by adding a variable, traumatic brain injury, using Cox proportional hazards regression for in-hospital mortality.

	Hazard ratio	95% confidence interval	*p*-value
Sex			
Male	Reference		
Female	0.76	0.63-0.91	0.003
